# Experience on Safety, Expulsion, and Complication of Intracesarean Post-Partum Intrauterine Copper Device

**DOI:** 10.7759/cureus.10647

**Published:** 2020-09-25

**Authors:** Soniya Vishwakarma, Vandana Verma, Mamta Singh, Nupur Mittal

**Affiliations:** 1 Obstetrics and Gynecology, Uttar Pradesh University of Medical Sciences, Saifai, IND

**Keywords:** intracesarean, ppiucd, missed thread, expulsion

## Abstract

Background

The post-partum period is one of the critical times when ovulation is highly unpredictable and couples often underestimate the likelihood of pregnancy. According to the World Health Organization, intrauterine copper device (IUCD) can be inserted within 48 hours post-partum, referred to as post-partum IUCD (PPIUCD). The objectives of the present study were to determine the safety and expulsion of intracesarean PPIUCD. This study also has an objective to determine the complications (undescended/missed thread, bleeding, pain, and infection) following intracesarean PPIUCD insertion among the women.

Materials and methods

This was a prospective interventional hospital-based study conducted in the Department of Obstetrics & Gynecology, Uttar Pradesh University of Medical Sciences, Saifai, Etawah, Uttar Pradesh, India, from November 1, 2016, to October 31, 2019. Women were followed up at six weeks and six months for various objectives.

Results

Intracesarean PPIUCD was inserted in a total of 1,586 patients, and 1,029 cases came for follow-up at six weeks and six months; thus, the concluded sample size was 1,029. The majority of patients were of 20 to 25 years of age, belonged to rural areas, and were having parity 2. The most common complaint was of undescended/missed thread in 22.2% women followed by bleeding (11.9%), expulsion (2.2%), pain (2%), and local infection (1.3%) at six weeks follow-up. At six months, the most common complaint was missed thread in 8.6% followed by bleeding (6.0%), pain (1.6%), expulsion (1.2%), and local infection (0.7%). There was no case of perforation. While 19.05% women wanted the removal of PPIUCD, but at the end of the study period, it was removed in a total of 11.27% cases due to various reasons.

Conclusions

PPIUCD is an effective tool to reduce the unmet need of contraception. This study showed that most of the women were satisfied with the intracesarean insertion of IUCD, indicating its important place within the basket of post-partum family planning methods.

## Introduction

India accounts for 17.85% of the world’s population [1] by adding around 25 million births every year in 2017. Global estimates have shown that effective usage of contraception can prevent 90% of maternal deaths related to unsafe abortions and 20% of overall obstetrics causes of mortality [2].

According to the National Family Health Survey 4, the current total unmet need for contraception is 12.9% and the unmet need for spacing is 5.7% [3]. The post-partum period is one of the critical times when ovulation is highly unpredictable and couples often underestimate the likelihood of pregnancy and expose women to the risk of unintended pregnancy. This presents with short interpregnancy interval and high fertility rate, contributing to high maternal and neonatal morbidity and mortality [4].

Various post-partum family planning (PPFP) methods available for the prevention of unintended and closely spaced pregnancies are lactational amenorrhea, barrier methods, progesterone-only preparations, and intrauterine copper device (IUCD) as temporary methods. According to the World Health Organization’s (WHO) Medical Eligibility Criteria (MEC), IUCD can be inserted within 48 hours post-partum, referred to as a post-partum IUCD (PPIUCD) [5]. PPIUCD insertion assures that woman is not pregnant and is convenient for women and service provider, especially when women have limited access to medical care and delivery may be the only time when they come into contact with health care providers.

We conducted this study to assess the safety, complications, and expulsion of intracesarean PPIUCD insertion within the rural tertiary-care hospital in India, which caters to an outsized heterogeneous population in its catchment basin. Though this topic seems older, still it is a new concept altogether for pregnant women of rural India.

The primary outcome measures of intracesarean PPIUCD in target women were safety (unusual vaginal discharge, infection, and irregular bleeding), expulsion, and complications.

## Materials and methods

This study was conducted in the Department of Obstetrics & Gynecology, Uttar Pradesh University of Medical Sciences, Saifai, Etawah, Uttar Pradesh, India, a rural tertiary care center. Intracesarean PPIUCD insertion was performed from November 1, 2016, to April 30, 2019, and each patient was followed up for six months; thus, the total duration of the study was from November 1, 2016, to October 31, 2019.

Study design

This is a prospective interventional hospital-based study. Approval from the Institutional Ethical Committee was taken before proceeding with this study.

All the patients who were included in the study belonged to a reproductive age group of 18 to 45 years, satisfying the WHO MEC-1 criteria [5,6] for PPIUCD insertion and with hemoglobin ≥ 9 g/dL.

Patients with prolonged rupture of membranes for more than 18 hours or chorioamnionitis or sepsis, unresolved post-partum hemorrhage, any structural abnormality of the uterus or a large fibroid distorting the uterine cavity, and coagulation disorder were excluded from the study.

All the patients who were planned for emergency or elective cesarean section and were meeting the inclusion/exclusion criteria were counseled for intracesarean IUCD insertion and written informed consent was taken from all the patients who were included in the study. The government supplied Multiload Cu 375 was used for intracesarean PPIUCD insertion.

For all the patients who were willing for PPIUCD insertion, basic demographic details were noted. History-taking, examination, and investigation were performed to consider her fitness for the PPIUCD as per the Post-partum IUCD Reference Manual [6] criteria. All the cases enrolled for PPIUCD insertion were given a particular family planning enrollment number.

Intracesarean PPIUCD was inserted in a total of 1,586 patients, and 1,029 cases came for follow-up at six weeks and six months; thus, the concluded sample size was 1,029.

Intracesarean insertion of IUCD (PPIUCD)

The IUCD was removed from the insertion sleeve and placed on the sterile ﬁeld. During cesarean delivery, after delivery of the baby, active management of the third stage of labor was performed, and after removal of the placenta, an examination of the intrauterine cavity was performed to rule out bleeding and uterine malformation. Now, the uterus was stabilized by grasping it at the fundus, and Multiload® Cu375 was held between the index finger and the middle finger and placed at the fundus of the uterus through the uterine incision. Care was taken not to include IUCD string while suturing the lower uterine segment.

A discharge card was given to the patients with information about the date of insertion and date of a post-partum follow-up visit and the patient was told to come for follow-up whenever any complaints are present, such as foul-smelling vaginal discharge, excessive bleeding, or lower abdominal pain associated with fever, or if there is suspicion that the device has been expelled.

Follow-up was conducted at six weeks and week months at the hospital, and history-taking and examination were performed to evaluate any complaint. TVS (transvaginal sonography) was performed in those women who had an undescended thread, suspicion of expulsion, or any other complaint.

## Results

Intracesarean PPIUCD was inserted in a total of 1,586 patients, and 1,029 cases were followed up at six weeks and six months; thus, the concluded sample size was 1,029.

The majority of patients (66.4% [n = 683]) who accepted and followed up for intracesarean PPIUCD were of 20 to 25 years of age. A total of 90% (n = 926) women were Hindus and 70.7% (n = 727) belonged to rural areas. About 30.4% (n = 312) and 34.7% (n = 358) women who were primary and secondary educated, respectively, responded well to counseling regarding long-acting reversible contraception. More than two-thirds, i.e., 72.1% (n = 742), of women belonged to low socioeconomic status and opted for a given choice of government-supplied IUCD (Table 1).

**Table 1:** Sociodemographic profile of cases

Sociodemographic factors		N = 1,029	Percentage
Age	21-25 year	683	66.4%
	26-30 year	314	30.5%
	31-35 year	32	3.1%
Residence	Rural	727	70.7%
	Urban	302	29.3%
Education	Uneducated	187	18.2%
	Primary	312	30.3%
	Secondary	358	34.7%
	Graduate	172	16.8%
Religion	Hindu	926	90.0%
	Muslim	103	10.0%
Socioeconomic status	Low	742	72.1%
	Middle	287	28%

In our study group, majority (49.9% [n = 514]) of patients were having parity 2; the details of parity-wise distribution are shown in Table 2.

**Table 2:** Distribution of cases according to parity

Parity	No. of women	Percentage
Primi para	342	33.3%
Parity 2	514	49.9%
Parity 3	159	15.4%
Parity ≥ 4	14	1.3%

**Figure 1:**
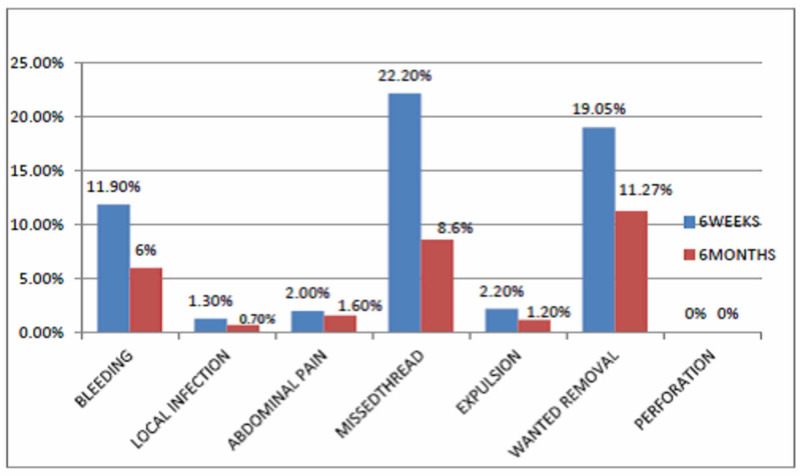
Complication at six weeks and six months follow-up Blue bar shows the six weeks follow-up data, whereas the red bar shows six months follow-up data.

All the patients were followed up at the hospital at six weeks and six months. The most common complication at six weeks were of undescended /missed thread in 22.2% (n = 228) women, which later reduced to 8.6% (n = 89) at six months followed by bleeding in 11.9% (n = 123), pain in 2% (n = 21), expulsion in 2.2% (n = 23), and local infection in 1.3% (n = 14) woman, all patients were counseled to continue with PPIUCD and given symptomatic treatment, only few women got PPIUCD removed at six weeks. At six months, the most common complaint was of missed thread in 89 (8.6%) women followed by bleeding in 6.0% (n = 61), abdominal pain in 1.6%, (n = 17), expulsion in 1.2% (n = 13), and local infection in 0.7% (n = 8) women. A total of 3.4% (n = 36) cases reported to the hospital with complaints of PPIUCD expulsion, 2.2% (n = 23) expelled within six weeks, and 1.2% (n = 13) expelled between six weeks to six months (Figure 1).

**Figure 2:**
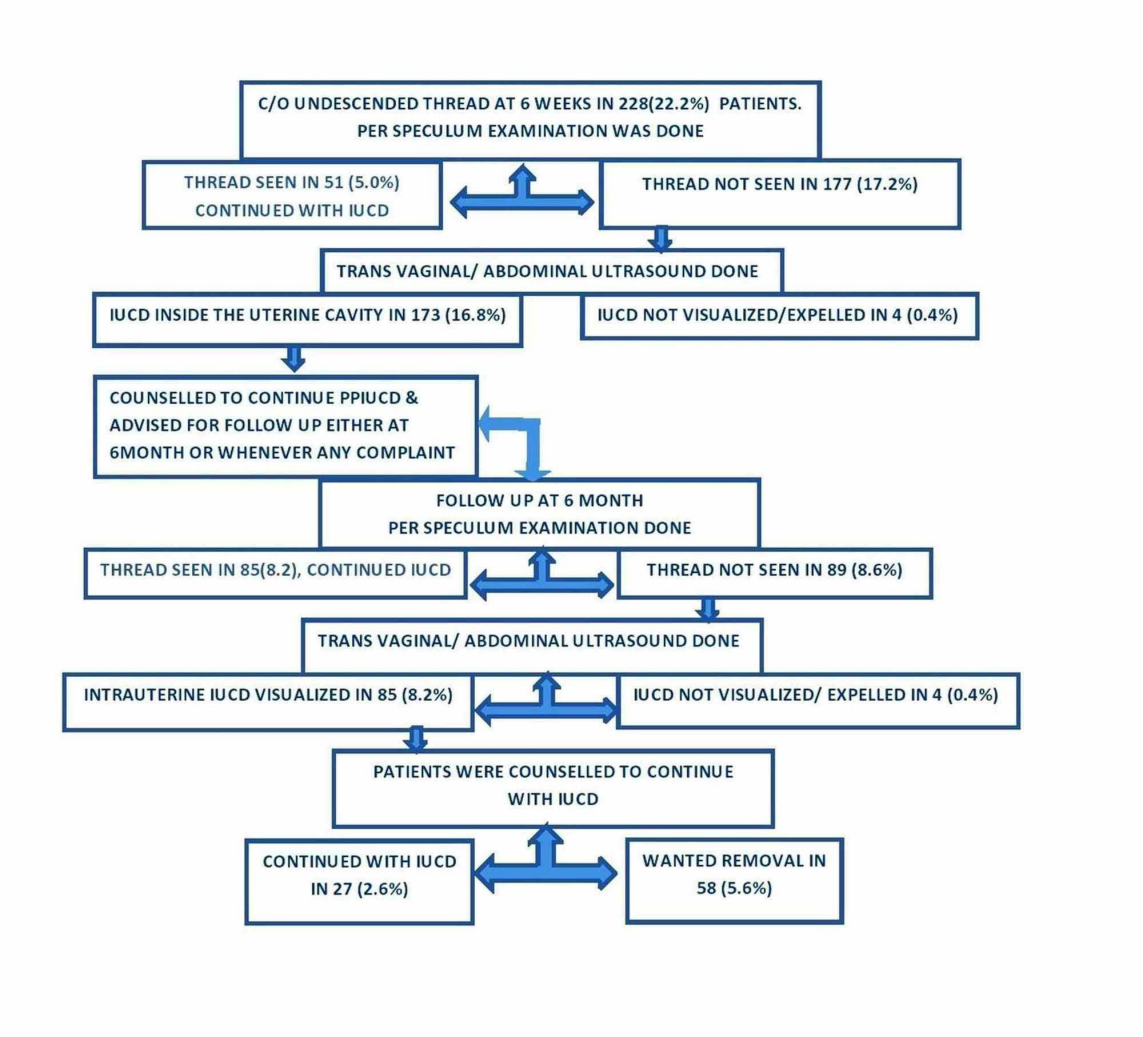
Fate of the undescended thread

 In hospital follow-up, 22.2% (n = 228) patients complained of undescended thread at six weeks; per speculum examination was performed and Multiload Cu375 thread was seen in 5.0% (n = 51) cases, but there was missed thread in 17.2% (n = 177) cases at six weeks. Transvaginal or abdominal ultrasound was performed in women with undescended thread, and IUCD was seen in the intrauterine cavity in 16.8% (n = 173) women, who were all counseled to continue with PPIUCD and for repeat follow-up at six months. At six months follow-up, per speculum examination was performed and IUCD thread was not seen in 8.6% (n = 89) women, and on ultrasonography, IUCD in place was seen in 8.2% (n = 85), but 5.6% (n = 58) women wished for IUCD removal for dissatisfaction due to missed thread (Figure 2).

During the study period, a total of 19% (n = 196) wanted PPIUCD removal, but after counseling, 7.7% (n = 80) continued with PPIUCD and it was removed in a total of 11.2% (n = 116) cases due to various reasons. A total of 17 women got IUCD removed at six weeks due to bleeding in 14 women and due to family pressure and other reasons in the remaining. All patients were counseled to continue with IUCD, but still 99 women got it removed at or before six months follow-up for various reasons, with the most common being missed thread in 5.6% (n = 58) followed by bleeding and family pressure, and few wanted to use other methods of contraception (Table 3).

**Table 3:** Removal of PPIUCD: causes PPIUCD, post-partum intrauterine copper device

Causes for removal		At six weeks	At/after six month	Total, N = 1,029 (%)
1.	Dissatisfied because of missed thread	-	58	58 (5.6%)
2.	Bleeding	14	17	31 (3.0%)
3.	Family pressure	2	13	15 (1.4%)
4.	Change over to other method	-	4	04 (0.4%)
5.	Others	1	7	08 (0.7%)
Total		17	99	116 (11.2%)

## Discussion

As lower section cesarean section (LSCS) rate is increasing for various reasons nowadays, women need an effective long-term reliable method of child spacing; termination of an unplanned pregnancy in patients with a previous scar is dangerous.

In rural India, where the literacy rate is low and women are mostly dependent on her family for hospital visits, we would like top-quality care contraception that needs a lesser number of follow-up visits with higher efficacy and lesser complications. The intrauterine contraceptive being long-acting reversible contraceptive fits well within these rural settings, but there are certain myths associated with IUCD in the society, which are required to be changed with motivation. Counseling helps best while a woman is either pregnant or in early labor or when she goes through the milestones and minor ailments of pregnancy or early labor, which is also the simplest time to motivate her for the role of birth spacing, especially in case of cesarean delivery. However, because women especially in rural India, generally, if not well motivated won't come for follow-up and are reluctant to acquire medical aid, to assess the safety of this method is of concern for us.

In our study group, maximum women were within the age bracket of 21-25 years (66.4%) followed by 26-30 years, which is analogous to reviews performed by Bansal et al. [7] and Haider et al. [8], which found that acceptance of PPIUCD was best within the age bracket of 21-25 years (39.33% and 44%, respectively). Jairaj and Dayyala [9] reported the mean age of acceptance as 23.70 years for post-partum copper T insertion, concluding that the acceptance of PPIUCD as contraception was more within the younger age bracket (≤30 years).

India may be a developing country where effective, safe, and future contraception with fewer side effects at low cost is important. The majority of women in our study belonged to the lower/middle class of socioeconomic strata. PPIUCD insertion is seen more in rural women (70.7%) as compared to urban (29.2%), and this is often probably because due to lack of transportation facilities from remote areas, unavailability of peripheral health services, and no prefixed ideas regarding IUCD. In a study conducted by Jakhar and Singhal [10], 54.5% of patients belong to rural areas and rest belonged to urban areas. Related social misbelieve within the area people are more in the urban areas. The majority of patients within the study were literate and completed secondary education (34.7%) followed by primary education (30.3%). Literacy made them understand the importance of follow-up visits. Jain et al. [11] showed that education level in women was more than primary level education (76.61%) and that 23.39% women were illiterate. Thus, education has a positive effect on contraceptive acceptance.

Out of 1,029 patients in this study, 49.9% were having parity 2 followed by parity 1 (33.3%), which was lesser than the study conducted by Jakhar and Singhal [10] in which 67.5% were gravida 2, mostly previous LSCS. Lowest acceptance was found in those belonging to parity 4 (1.4%), which was almost similar to the findings of Goswami et al. [12] and Rani et al. [13], who found that women with parity 2 had a high acceptance (48% and 51.6%, respectively). On the contrary, Bansal et al. [7] and Maluchuru et al. [14] found a better acceptance in parity 1, which was 36.0% and 31.46% respectively.

Within our study, PPIUCD follow-up showed that 3.4% women had expulsion, which was similar to a study conducted by Yadav et al. [15], who found the expulsion rate at four to six weeks interval as 3.12%. A study conducted by Jairaj and Dayyala [9] reported a complete expulsion rate of 6.8%. A study by Rani et al. [13] reported a really low expulsion rate of 2.74%.

Visibility/feeling of IUCD thread is vital to make sure that the IUCD is in situ, though sometimes a woman cannot feel the thread even when IUCD is inside the uterine cavity. The commonest complaint was undescended IUCD thread (22.2%) at six weeks follow-up at the hospital; when per speculum examination was performed, thread was seen in 5% of cases, and IUCD expelled in 0.4%, thus the missed thread was absolutely missed in 16.8%, which was similar to the finding by Bansal et al. [7] within which missed thread rate was reported to be 16.2% at six weeks follow-up. Barala et al. [16] reported missing strings in 8% in their study at six weeks follow-up. In women with missed thread at six weeks, TVS or abdominal ultrasound was performed and IUCD was seen inside the uterine cavity in 16.8%. All the patients with missing strings were counseled to continue with PPIUCD and to review again at six months; per speculum examination was performed again, and the thread was not seen in 8.6% women at six months, which was much lower than that in the study conducted by Jain et al. [11], in which cumulative incidence of the missed thread was 26.5% at six months to one year follow-up. Good counseling may lessen the number of patients who get IUCD removed during their follow-up due to missing thread.

In our study, 11.9% patients had a complaint of bleeding at six weeks, which responded well to hemostasis, leaving only 6% cases with this complaint at six months. In a study conducted by Jain et al. [11], significant bleeding was seen in 10.7% women, and excessive bleeding settled with hemostasis within one to three months.

Other complaints were abdominal pain (2.0%) and local infection (1.3%) at six weeks follow-up, which responded well to antibiotic therapy. At six months, only 1.6% and 0.7% had abdominal pain and local infection, respectively, which was much lower than that reported in the study conducted by Jain et al. [11], in which pain abdomen was seen in 8.6% of patients. In a study of Jakhar and Singhal [10], 14% of cases complained of abdominal pain at six months. In a study conducted by Jairaj and Dayyala [9], the main reported complications were pain abdomen (17.14%) and bleeding (14.28%).

Among those with complications, the majority of patients responded well to counseling and symptomatic treatment and continued with PPIUCD. While 11.2% of women still wanted removal of PPIUCD, which was similar to a study conducted by Jain et al. [11], where removal of IUCD was performed in 14.5% only and increased bleeding P/V (per vaginal) was the commonest cause. In our study, the commonest cause for removal was dissatisfaction due to missing thread (5.6%), bleeding (3.0%), and family pressure. This was less in a study conducted by Jairaj and Dayyala [9], where the most common reason (40%) for the removal of IUCD was the inclination of other methods. in a study by Rani et al. [13], 5.48% of women came for the removal of IUCD, and therefore the main reason behind removal was pressure from family and lower abdominal pain.

In our study, the continuation rate was 88.8%. A high continuation rate of 98.8% in women having no side effects and a continuation rate of 64.28% in women having some side effects were seen by Barala et al. [16]. The high continuation rate of PPIUCD in our study was similar to that in the study conducted by Jain et al. [11], as 85.5% of patients continued to retain their IUCD after six months to one year, and Jakhar and Singhal [10], where the continuation rate after six months was 89.5%.

## Conclusions

The patients were willing to continue with the device, indicating a high level of satisfaction, and were willing to recommend this method to others additionally. Besides, some women requested removal for a variety of reasons, with the most common being a missing thread, bleeding, and family pressure.

Thus, within the post-partum period, PPIUCD is an efficient tool to scale back the unmet need for contraception. This study showed that the majority of women were satisfied with the intracesarean insertion of IUCD, indicating its important place within the basket of PPFP methods.
